# Socioeconomic status and race/ethnicity independently predict health decline among older diabetics

**DOI:** 10.1186/1471-2458-11-684

**Published:** 2011-09-02

**Authors:** Emily J Nicklett

**Affiliations:** 1 School of Social Work, University of Michigan, 1080 South University Avenue, Ann Arbor, MI 48109-1106, USA

## Abstract

**Background:**

There are pervasive racial and socioeconomic differences in health status among older adults with type 2 diabetes. The extent to which racial/ethnic and socioeconomic disparities unfold to differential health outcomes has yet to be investigated among older adults with diabetes. This study examines whether or not race/ethnicity and SES are independent predictors of steeper rates of decline in self-rated health among older adults in the U.S. with type 2 diabetes.

**Methods:**

The study population was a subset of diabetic adults aged 65 and older from the Health and Retirement Study. Respondents were followed up to 16 years. Multilevel cumulative logit regression models were used to examine the contributions of socioeconomic indicators, race/ethnicity, and covariates over time. Health decline was measured as a change in self-reported health status over the follow-up period.

**Results:**

Relative to whites, blacks had a significantly lower cumulative odds of better health status over time (OR: 0.61, p < .0001). Hispanics reported significantly lower cumulative odds better health over time relative to whites (OR: 0.59, p < .05). Although these disparities narrowed when socioeconomic characteristics were added to the model, significant differences remained. Including socioeconomic status did not remove the health effects of race/ethnicity among blacks and Hispanics.

**Conclusions:**

The author found that race/ethnicity and some socioeconomic indicators were independent predictors of health decline among older adults with diabetes.

## Background

Diabetes is projected to increase in age-adjusted prevalence 2.2 times among those aged 65-74 years and 4.5 times among those 75 years and above from 2005-2050 [1]. Among older adults, diabetes accelerates cognitive decline, physical disability, and other limitations [2,3]. The burden of diabetes, however, is not evenly spread in the U.S. population. Diabetes is more prevalent among disadvantaged groups and communities, predicted by race and socioeconomic position [4,5]. Disadvantaged groups also suffer more from complications associated with the disease; disparities have been found in excess mortality, functional status, and cognitive functioning [6-8]. Diabetes could operate as a mechanism through which health disparities between socially advantaged and the socially disadvantaged populations are exacerbated.

Racial and ethnic disparities in health have long been documented in public health literature. The underlying mechanisms include differing health behaviors, medical decision-making, quality and access to care, and cumulative effects of discrimination [9-13]. These mechanisms are intertwined with socioeconomic status, which has been linked to health generally through, for example, relative and absolute disadvantage, neighborhood effects and residential segregation, and access to care [14-16].

Previous longitudinal research has found that spells of wealth and poverty predict subsequent health outcomes [17,18]. The majority of research studies found that race/ethnicity is no longer predictive of long-term health after socioeconomic factors are included in the models [8-20]. Research examining general health decline and disablement has come to similar conclusions when taking incident morbidity into account [21-23]. In contrast, some longitudinal research has also found that racial/ethnic status remains a strong predictor of health status as people age (after controlling for socioeconomic characteristics). For example, several studies found strong interaction effects between race/ethnicity and sociodemographic characteristics [24-26]. Despite these conflicting findings, few studies have examined whether race/ethnicity and socioeconomic status independently predict health outcomes. The few studies that examine this question, for example, use cross-sectional data, examine different chronic diseases, or examine mortality as an outcome [7,8,10].

This study examines race/ethnicity and socioeconomic status as predictors of health decline over time among older adults with diabetes. This is the first study to examine the longitudinal health outcomes of diabetics by race/ethnicity and socioeconomic status among diabetics in a population-based study. Race/ethnicity and socioeconomic indicators are analyzed separately and together to better examine these layered forms of disadvantage. Further, this study allows for different measures of SES and other predictors over time contributing to the health disparities literature by addressing what aspects of socioeconomic position influences health decline. By providing a more sophisticated analysis that accounts for temporal precedence, such dynamic research could provide insight on why findings in previous literature are inconsistent or lead to mixed results.

The present research contributes to social epidemiological and public health research. The primary aim is to examine how socioeconomic characteristics and race/ethnicity predict the rate of decline of health status among older, chronically ill adults. The dynamic nature of health is captured through analysis of both time-constant and time-varying characteristics. Finally, it examines whether these relationships hold after controlling for individual-level health covariates.

The findings could have implications for policy and practice because of the high degree to which diabetes can be prevented and controlled [27]. This analysis focuses on older adults with diabetes for several reasons. Diabetes is increasingly prevalent, particularly among disadvantaged populations. Health disparities research investigating longitudinal health outcomes has flourished in the last decade; however, additional insight is needed for the social determinants of disparities in distinct population groups (such as older adults with diabetes). Further examining this distinct yet growing population group also can contribute to knowledge in the epidemiological, health services and health delivery fields. This study was approved by the University of Michigan Institutional Review Board.

## Methods

### Study Population

The Health and Retirement Study (HRS) is a national, population-based study that has tracked individuals and households since 1992 [28]. The first cohort (1992) included 12,654 adults born from 1931-1941. Adjusting for mortality, the response rates have remained above 84% in the seven subsequent biennial waves.

### Analytic Sample

Longitudinal data spanning 16 years is examined. Data were collected biennially, with a maximum of 8 repeat observations. Data are drawn from the RAND (2008) combined data files. Item missing data is imputed according to RAND's criteria [29]. The analytic sample consists of 2,494 diabetic individuals of the 3 ethnic groups examined (white, black, or Hispanic) who participated in at least 3 survey waves (those who became diabetic are only included in the waves after which they report having diabetes). The majority of these respondents (2,379) were included in the analytic sample at baseline. As this study focuses on older adults, the sample was restricted to respondents the age of 65 years or greater at the time of a given wave's interview. The study population was restricted to focus on processes by which health declines among an ever-growing segment of our population--chronically ill older adults--and how these processes intersect with race/ethnicity and socioeconomic statuses. The age 65 was selected as a minimum to reduce age-effects introduced by specific age-eligible programs and transitions such as retirement, Medicare and Social Security, which could have different effects for health status according to racial/ethnic or socioeconomic group. Observations are in person-year due to the longitudinal tracking nature of the study. The analytic sample includes 2,494 respondents, but there are a total of 19,061 observations, as respondents participated in multiple waves.

### Outcome measure

Health decline was assessed by changes in self-reported health over time. Health status is examined in cumulative odds of reporting an incrementally higher health value. Values were recoded to reflect 5 (excellent), 4 (very good), 3 (good), 2 (fair), and 1 (poor).

### Race/Ethnicity measures

Baseline *race/ethnicity *categories were assigned by looking at reports from all waves of data for race. Respondents initially identified as White/Caucasian, Black/African American, or Other. When asked whether Hispanic or non-Hispanic, respondents were categorized as Hispanic according to the first non-missing value answered. Therefore, three mutually exclusive categories of non-Hispanic white, non-Hispanic black, and Hispanic were created that remain consistent across waves.

### Socioeconomic measures

A baseline measure of *education *was used. The cross-wave highest degree categorical variable is assigned by utilizing the first non-missing value across survey waves. Three distinct categories were generated, including less than high school (some high school or less), high school (high school or GED), and some college or more (AA, BA, or graduate-level education). The time-varying measure *total household income *(in $100 s) is the sum of all income in the household. RAND adjusted for slight variations in questions across waves [29]. Smaller increments were used to examine household income than household assets as the former has a smaller range of values. The time-varying measure *total household assets *(in $1000 s) is the net value of total wealth minus all debt, including primary and secondary residences, and assets. Debts (including mortgages and other debts) are subtracted from positive assets to equal the final value. Education, household income, and household assets were generally not highly correlated: the highest correlation was 0.5 (between household assets and household income). Analyses were also tested with only income (in which income was slightly significant) and with only assets (in in which assets were slightly more significant) - as well as subsequent analyses - suggested that this high correlation is not driven by a variable omitted from this analysis. Income and asset variables were also tested in logged and quadratic forms (with similar levels of significance). Initial values were retained for improved interpretation of the coefficients.

### Covariates Analyzed

Multivariate models controlled for health and socio-demographic covariates. These control variables included time-varying *body mass index *(*BMI*) and whether or not the respondent had *private health insurance *(in addition to Medicare). Time-varying *BMI *was calculated as weight divided by the square of height. As older adults often do not maintain their height, height measures are updated by wave when available. When updated height information was not available, height from previous waves was carried forward for missing cases. Changes in BMI over time suggests individual trends in adhering to practices that are typical of a diabetes regimen, such as engaging in physical activity and eating a healthful diet. The variable *private health insurance *(at baseline) indicates whether or not the respondent received insurance from his or her current or prior employer (or spouse's employer) in addition to public insurance such as Medicare [29]. If a respondent received private health insurance but did not in a subsequent wave, then the observation was replaced with a negative response. Baseline measures of *gender *and number of *chronic illness comorbidities *(in addition to diabetes) were also included. The baseline measure of *chronic illness comorbidities *(*comorbidity*) was calculated by summing the maximum number of up to five selected chronic illnesses at baseline: high blood pressure, cancer, lung disease, heart disease, stroke, psychiatric problems, and arthritis. Models also adjusted for baseline *working status*. Respondents were posed the question "Are you currently working for pay?" Missing data were imputed by RAND based on related questions in some waves (i.e. "are you working now?"). Working status could relate negatively or positively to one's position in society. For example, individuals might have to be physically healthy enough to work. Work could also be indicative of financial well-being (the option to work) or of financial hardship (the necessity to work). Benefits of work could include physical activity, social engagement, and cognitive exercise, while negative aspects could include stress or physical demands/strain. Finally, models adjusted for time-varying *age *in years.

### Analytic Strategy

Multilevel modeling was used to examine the relationships between socioeconomic status and race/ethnicity with long-term health decline. Three multilevel cumulative logit regression models were used [30-32]. The first two models examine the extent to which long-term health outcomes are predicted by socioeconomic status and race/ethnicity, respectively (controlling for health and sociodemographic covariates). The third model incorporates socioeconomic status measures and race/ethnicity, as well as covariates. Together, the models enable the analysis of the relationship between race/ethnicity and health as well as socioeconomic status and health.

The multilevel cumulative logit regression model, a specific form of multilevel ordered logit analysis, is appropriate due to the ordinal rank if the dependent variable from 1 (poor) to 5 (excellent), which considers floor, ceiling effects, and skewness more than does an OLS regression model. This model takes ordering of response categories (from poor to excellent health) into consideration when estimating how predictor variables relate to probabilities of a given response, has fewer parameters than multinomial logit regression models, and is therefore more parsimonious [33].

According to Heeringa and colleagues, the estimated coefficients from the multilevel cumulative logit regression models provide information about the relationships between the cumulative logits and the relationships of response and predictors [33]. For example, positive values suggest that relative to the reference category of poor health, the distribution of ordinal responses is shifted above that distribution. The resulting coefficient is an individual-specific proportional odds ratio. The ratio is interpreted as the odds of change from one interval of health to another over the entire study period.

This model allows the slope and intercept parameters of self-reported health status to vary across individuals and over time (as a random slope, random intercept model), so that they become dependent variables in the level two (or person-level) model, where individual characteristics are included as predictors. The multilevel cumulative logit procedure is appropriate for this analysis due to the ability to analyze changes within and between individuals and groups over time, taking into consideration the dynamic role of behaviors and circumstances over the life course. This model assumes that the random intercept and random slope follow a normal distribution and are independent across respondents. The proportional odds assumption was not violated. All were conducted using Stata. Significance is tested at *P *< .05.

Model 1: The first model examines racial/ethnic differences in the steepness or rate of health decline. Racial/ethnic variables (white, black, and Hispanic) and all health-related and sociodemographic covariates, excluding socioeconomic measures were included in the model.

Model 2: The second model examines socioeconomic differences in the steepness of health decline. Specifically, it examines whether those with lower levels of education experienced successively steeper rates of health decline, whether increases in income in are associated with steadier rates of decline, and whether increases in wealth are associated with steadier rates of decline of self-rated health. Finally, socioeconomic measures and all health-related and sociodemographic covariates are included. Race/ethnicity are excluded from the model.

Model 3: The final model tests whether any racial/ethnic and socioeconomic differences suggested by prior models remain when simultaneously adjusted in the model. This model includes all measures and covariates.

## Results

### Descriptive Statistics

Table 1 shows that self-rated health varies by socio-demographic, socioeconomic, and health characteristics. Table 2 shows that socioeconomic characteristics also differ according to race/ethnicity. Over the study period, 71% remained living. Blacks were more likely to experience mortality on average and Hispanics were less likely to experience mortality during the duration of the study period.

**Table 1:** Distributrion of Pooled Sample Characteristics by Self-Rated Health among Older Adults with Type 2 Diabetes, 1992-2006

	Unwgtd. N	1 (Poor)	2	3	4	5 (Excellent)
Race/ethnicity						

White (%)	1846	27.25	29.69	27.19	13.22	2.65

Black (%)	467	31.48	38.12	21.41	7.49	1.5

Hispanic (%)	181	27.07	43.65	22.65	5.52	1.1

Education at baseline						

Less than high school (%)	1907	34.86	35.83	19.95	7.59	1.77

High school/GED (%)	357	25.06	28.94	30.88	12.92	2.20

Some college or more (%)	229	18.60	29.86	30.38	17.58	3.58

Mean household income per year (in $100 s) ^†^	10342	206.91	252.56	365.27	371.26	421.14

Mean household assets (in $1000 s)^†^	10342	13.88	18.87	33.15	39.61	35.52

Mean BMI ^†^	10207	26.27	26.91	27.09	26.68	26.79

Health insurance at baseline						

No private health insurance (%)	1317	36.49	32.43	25.68	2.70	2.70

Private health insurance (%)	957	24.00	37.33	28.00	8.00	2.76

Sex						

Male (%)	1114	27.02	31.60	26.84	11.94	2.60

Female (ref) (%)	1380	28.84	32.83	24.93	11.30	2.10

Comorbidities at baseline (in addition to diabetes)						

0 (%)	115	6.09	26.96	40.00	18.26	8.70

1 (%)	310	16.13	27.74	31.29	22.26	2.58

2 (%)	639	19.41	31.92	31.46	13.93	3.29

3 (%)	722	28.95	35.32	23.82	9.83	2.08

4 (%)	473	39.75	33.19	19.24	7.40	0.42

5 or more (%)	234	51.28	30.77	15.38	1.71	0.85

Working Status at baseline						

Not working (%)	2382	28.76	32.62	25.19	11.29	2.14

Working (ref) (%)	104	9.62	25.00	39.42	19.23	6.73

Mean age^†^	10340	80.47	80.98	80.78	80.73	80.83

^†^Indicates variables that vary across waves. Variables measured at baseline have a maximum of 2494 observations. Variables that vary across waves have pooled respondent samples, with a maximum of 19061 observations from 1992-2006.

**Table 2:** Distributrion of Pooled Sample Characteristics by Race/Ethnicity among Older Adults with Type 2 Diabetes, 1992-2006

	Unwgtd. N	White	Black	Hispanic
Education at baseline				

Less than high school (%)	1907	76.48	87.58	92.57

High school/GED (%)	357	14.18	7.67	4.00

Some college or more (%)	229	9.34	4.74	3.43

Mean household income per year (in $100 s) ^†^	10342	344.41	171.59	143.32

Mean household assets (in $1000 s)^†^	10342	28.41	6.07	6.79

Mean BMI ^†^	10207	27.32	28.17	27.63

Health insurance at baseline				

No private health insurance (%)	1317	47.11	33.25	18.13

Private health insurance (%)	957	52.89	66.75	81.88

Sex				

Male (%)	1114	47.47	36.57	40.57

Female (ref) (%)	1380	52.53	63.43	59.43

Comorbidities at baseline (in addition to diabetes)				

0 (%)	115	4.38	4.06	5.71

1 (%)	310	11.88	11.51	16.00

2 (%)	639	24.83	28.67	26.29

3 (%)	722	28.64	30.70	29.14

4 (%)	473	19.94	16.93	16.57

5 or more (%)		10.34	8.13	6.29

Working Status at baseline				

Not working (%)	2382	90.65	68.52	51.03

Working (ref) (%)	104	9.35	31.48	51.03

Mean age^†^	10340	77.58	78.12	77.41

^†^Indicates variables that vary across waves. Variables measured at baseline have a maximum of 2494 observations. Variables that vary across waves have pooled respondent samples, with a maximum of 19061 observations from 1992-2006.

### Race/Ethnicity

As shown in Model 1 in Table 3, blacks had a significantly lower cumulative odds of better health status over time than whites (OR: 0.61, p < .0001). Hispanics reported significantly lower cumulative odds better health over time relative to whites (OR: 0.59, p < .05). In Model 3 (which controls for socioeconomic characteristics), the effects narrowed (OR: 0.70, 0.68, p < .05) from the previous model, but significant differences remain. Including socioeconomic status did not remove the health effects of race/ethnicity among blacks and Hispanics, although the effect did weaken in magnitude and statistical significance.

**Table 3:** Separate Multilevel Cumulative Logistic Regression Models Predicting Self-Reported Health among Older Adults with Type 2 Diabetes, 1992-2006

	Model 1	Model 2	Model 3
	**OR (95% CI)**	**OR (95% CI)**	**OR (95% CI)**

Race/ethnicity (ref: White)			

Black	0.614 (0.473, 0.796)	-	0.701 (0.543, 0.905)

Hispanic	0.592 (0.407, 0.860)	-	0.684 (0.474, 0.988)

Education at baseline (ref: less than high school)			

High school/GED	-	1.046 (0.711, 1.539)	1.026 (0.699, 1.506)

Some college or more	-	0.644 (0.460, 0.900)	0.666 (0.477, 0.930)

Household income per year (in $100 s) ^†^	-	1.074 (0.947, 1.218)	1.068 (0.942, 1.210)

Household assets (in $1000 s)^†^	-	1.391 (1.167, 1.658)	1.342 (1.127, 1.596)

BMI ^†^	1.012 (0.993, 1.080)	1.012 (0.993, 1.030)	1.013 (0.995, 1.032)

Health insurance at baseline	1.481 (1.208, 1.815)	1.417 (1.161, 1.729)	1.361 (1.115, 1.662)

Sex (ref: female)			

Male	0.964 (0.795, 1.170)	1.006 (0.830, 1.219)	1.019 (0.842, 1.234)

Comorbidities at baseline	0.534 (0.476, 0.599)	0.552 (0.495, 0.616)	0.547 (0.490, 0.611)

Working at baseline	2.746 (1.708, 4.417)	2.376 (1.485, 3.802)	2.370 (1.486, 3.780)

Model Characteristics			

Log Likelihood	-2942.620	-2928.521	-2923.391

Condition Number	2811.638	2865.067	2861.017

Level 1 and Level 2 units	2233, 2151	2232, 2141	2232, 2141

Variance of random effects (Level 1, Level 2)	1.355, -0.573	1.241, -0.542	1.198, -0.534

^†^Indicates variables that vary across waves.

### Socioeconomic Characteristics

Having a high school degree or equivalent was not predictive of health decline. Higher education predicted worse subsequent health: respondents with some college or more had 0.64 cumulative odds of better health status over time (p < .05) relative to their counterparts who did not complete high school. Income was not a significant predictor, but assets were significantly associated with improved cumulative odds of better health. When race/ethnicity is included in Model 3, the relationship and significance of the sociodemographic characteristics remain but again weaken in magnitude. For example, the coefficient for household assets decreases slightly from 1.39 to 1.34 (p < .001). Level of education was a significant predictor in the opposite direction predicted.

## Discussion

This research examined the complex relationships between health status, race/ethnicity, and socioeconomic status among older diabetics in the US, considering key individual level variables raised as potential explanations for health disparities in previous research. In this study, race/ethnicity was a significant predictor of cumulative odds of better health, with whites faring better than Hispanics and blacks. Although the cumulative odds did decrease while adding socioeconomic variables to the analysis, race/ethnicity remained a strong and significant predictor of health decline. While these findings challenge previous research that found that racial/ethnic differences were no longer significant, it supports the findings of studies that found race/ethnicity to remain a significant predictor [24-26].

The findings that whites had steadier rates of decline than did blacks and Hispanics among an older adult, chronically ill population from the age of 65 suggest that even with access to diabetes-related health care treatments through Medicare and other age-associated programs, the health gaps between whites and non-whites grow over time. These factors remained significant while controlling for private health insurance and body mass index.

Some socioeconomic characteristics were protective against health decline over time, as found in previous research [17,18]. These relationships remained significant controlling for BMI and health insurance. The contributions of wealth, income, and education vary at different points of the life course to the onset or progression of a chronic disease. The finding that higher levels of education are significantly associated with more rapid rates of decline also warrants further study. Previous longitudinal research has suggested that education has less to do with disease outcomes, but more with the initial structures of ascribed statuses within a society relative to other socioeconomic measures [34,35]. In sum, race/ethnicity and household assets remain significant predictors of health status change over time when examined together. This finding suggests that social disadvantage--in terms of wealth and race/ethnicity--heightens the risk of worse long-term health outcomes among diabetics. Health disparities by race/ethnicity as well as by socioeconomic status must be addressed by policies or programs aiming to improve long-term health outcomes of aging, chronically ill populations.

## Conclusions

These findings provide a unique contribution to research on aging, chronic disease, and health disparities. Additional analyses are needed to gain greater depth into the causal relationships between these structurally linked characteristics and health status. Such analyses could focus of mediating factors (such as health insurance or health behaviors). Future analyses should also focus on subgroups with differing racial/ethnic and socioeconomic characteristics over time. It is necessary to further examine the mechanisms and processes underlying these divergent paths of health. The mechanisms underlying these divergent paths should be further explored to address how and why individuals experience disadvantage and if this accumulates in health disadvantage over time through racism, health discrimination, or segregation among older adults [13,16,36,37]. Further, access to private insurance in addition to Medicare could represent greater continuity of care which could be associated with better self-care behaviors, outcomes, or earlier disease discovery among diabetics [38]. Additional characteristics that should be examined in future studies include the stress, neighborhood characteristics, and quality of care over the life span.

The study has several limitations. Health is a complex state, not limited to self-reported health status. The study relies primarily on self-reported data, which could introduce bias of differential expectations by group. This analysis does account for differential expectations partially by examining changes among the same individuals over time; however, it is feasible that the individuals change their reference categories over time as their social position or health status also changes, which might introduce bias [39].

The population to which these findings can be generalized are older adults with diabetes in the US who have survived to the age of 65. This analysis follows time, rather than focusing on the transitions of age or cohort-specific changes (e.g. retirement, Medicare insurance), which is experienced differently by social position. As with most longitudinal studies of older adults, subject mortality could result in underestimated associations. Mortality is related to worse prior health reports and is more common among blacks and persons with low education in this sample. Previous studies have also found that HRS mortality is significantly more likely among non-whites, which might make the race/ethnicity estimates somewhat conservative [40]. Subjects must have also survived until the age of 65 to participate in the study, which exposes the different segments of the original HRS sample to selective mortality disproportionately. Mortality during the follow-up period among participants with worse health could also result in underestimated associations. This analysis therefore does not exclude mortality from the estimation of the natural course of diabetes.

Finally, the relationship between health and social characteristics is multi-faceted. More research is needed that focuses on intersectionalities, suggesting that the disadvantage is not simply additive or interactive, but could be multiplicative. Future analyses are necessary to better understand how social forces fundamentally shape illness experiences, outcomes, and how advantage and disadvantage unfold into and within chronic illness in the remainder of the life span.

## Competing interests

The author declares that they have no competing interests.

## Authors' contributions

EN conceived of the study, participated in the design of the study and performed the statistical analysis. EN drafted the manuscript and conducted all revisions.

## Pre-publication history

The pre-publication history for this paper can be accessed here:

http://www.biomedcentral.com/1471-2458/11/684/prepub
